# Satisfaction with online learning in the new normal: perspective of students and faculty at medical and health sciences colleges

**DOI:** 10.1080/10872981.2021.1920090

**Published:** 2021-05-11

**Authors:** Wiam Elshami, Mohamed H. Taha, Mohamed Abuzaid, Coumaravelou Saravanan, Sausan Al Kawas, Mohamed Elhassan Abdalla

**Affiliations:** a Medical Diagnostic Imaging Department, College of Health Science, University of Sharjah, Sharjah, United Arab Emirates; b College of Medicine and Medical Education Center, University of Sharjah, Sharjah, United Arab Emirates; c College of Medicine, University of Sharjah, Sharjah, United Arab Emirates; d Oral and Craniofacial Health Department, College of Medicine, University of Sharjah, Sharjah, United Arab Emirates; e School of Medicine, University of Limerick, Limerick, Ireland

**Keywords:** E-learning, online learning, student satisfaction, faculty satisfaction, health profession education, COVID-19

## Abstract

Online learning has become the new normal in many medical and health science schools worldwide, courtesy of COVID-19. Satisfaction with online learning is a significant aspect of promoting successful educational processes. This study aimed to identify factors affecting student and faculty satisfaction with online learning during the new normal. Online questionnaires were emailed to students (n = 370) and faculty (n = 81) involved in online learning during the pandemic. The questionnaires included closed- and open-ended questions and were organised into two parts: socio-demographic information and satisfaction with online learning. Descriptive statistics were used to analyse the responses to the satisfaction scales. Students’ and faculty responses to the open-ended questions were analysed using the thematic analysis method. The response rate was 97.8% for students and 86.4% for faculty. Overall satisfaction among students was 41.3% compared to 74.3% for faculty. The highest areas of satisfaction for students were communication and flexibility, whereas 92.9% of faculty were satisfied with students’ enthusiasm for online learning. Technical problems led to reduced student satisfaction, while faculty were hampered by the higher workload and the required time to prepare the teaching and assessment materials. Study-load and workload, enhancing engagement, and technical issues (SWEET) were the themes that emerged from the thematic analysis as affecting student and faculty satisfaction. Adopting a combination synchronous and asynchronous approach, incorporating different applications to engage students, and timely feedback are imperative to increasing student satisfaction, while institutional support and organisational policy could enhance faculty satisfaction.

## Background

Due to safety measures as a result of COVID-19, online learning has become a useful and practical tool for curriculum delivery worldwide [1,2]. Several advantages of online learning for learners have been reported in the literature, including easy accessibility to knowledge, proper content delivery, content standardisation, personalised instruction, self-pacing, interactivity and increased convenience [3]. During the COVID-19 pandemic, online learning has helped universities keep their doors open for students during lockdown to decrease the spread of the disease [1,2].

Although online learning is the only available solution during the COVID-19 pandemic, student and faculty satisfaction is crucial for a successful and effective learning process. Student and faculty satisfaction can be define as attitude resulting from an evaluation of educational experience, facilities and services [4]. Faculty satisfaction is defined as the perception of the online teaching process as efficient, effective and beneficial for both students and faculty [5]. On the other hand, student satisfaction is related to the value of the learning experiences [6].

The definition of satisfaction in online learning is complex and multidimensional and includes many factors, such as communication, student participation in online discussions, flexibility, workload, technology support, instructor pedagogical skills, and feedback [7,8]. Satisfaction with online learning is based on three learning theories: social cognitive theory, interaction equivalency theorem, and social integration theory [9–11]. Students construct knowledge in a social context while interacting with others, engaging in activities, and receiving feedback [9]. Students’ interactions with other students, instructors and content play a significant role in satisfaction. Therefore, satisfaction with the learning experience increases as multiple types of interactivity are used within the learning context [10]. Engaging students in formal extracurricular activities in addition to their academic programme improves student satisfaction. Informal faculty–peer social interaction is also valued in the learning process [11].

There is a growing body of literature showing that satisfaction has a positive relationship with student engagement and academic performance [12,13]. The quality of learning is based on faculty and student satisfaction along with learning effectiveness, access and institutional cost-effectiveness [14,15]. A previous study reported no significant differences between well-designed online and face-to-face learning [16]; however, some studies have found that participants were more satisfied with face-to-face teaching [17]. Other studies have reported that measuring student satisfaction in online learning is a significant aspect of successfully promoting educational processes for institutions, faculty and learners [18,19].

Factors affecting student and faculty satisfaction with online learning can be categorised into three main categories: faculty, interactivity, and technology [15,20] and students, instructor, and institution [21], respectively. Student and faculty satisfaction are interrelated, as student satisfaction is affected by interaction and technology, which require more effort from faculty to engage the students online besides the necessity of adequate techno-pedagogical skills [22]. Satisfaction across genders is a contradictory issue; while a study found that there are no differences between genders in online learning [23], another study found that female students were more satisfied with online learning than male students [24].

COVID-19 came abruptly with little or no preparation in place in many countries. The educational system during the COVID-19 era is characterised by a ‘new normal’. The term ‘new normal’ is described in the Urban Dictionary (2009) as a situation that occurs after an intense change. It was first used in the business field and other contexts to describe previously atypical life situations that have become typical [25]. Online learning has been used as an adjunct method to augment the classical approach to teaching. The sudden transition from face-to-face teaching to 100% online learning is courtesy of COVID-19. Numerous studies have measured either student or faculty satisfaction with online learning before COVID-19 [26–28]. To the best of our knowledge, no study has simultaneously measured both faculty and student satisfaction during the COVID-19 pandemic. Therefore, this study aimed to identify factors affecting student and faculty satisfaction with online learning during the COVID-19 pandemic.

## Methods

A cross-sectional study was conducted at Medical and Health Sciences Colleges between April and May 2020. The university had shifted suddenly during COVID-19 lockdown to deliver the curricula completely online using synchronous and asynchronous sessions utilising Blackboard and Microsoft Teams. Several training workshops and manuals were provided to both faculty and students. The university also established 24/7 technical support for both faculty and students.

An online questionnaire, along with the study information sheet and consent form, was sent to the expected participants in the Medical and Health Sciences Colleges. Participants were informed that participation was voluntary and that they could withdraw from the study at any time without consequence.

The sample size was 370 students and 81 faculty and was calculated using the formula of a finite population; the margin of error was set as 5%, and the confidence level was 95%. We used pre-validated questionnaires to measure student and faculty satisfaction with online learning [20,21].

The questionnaire was organised into two parts – socio-demographic information and satisfaction with online learning – using different satisfaction scales. The students’ satisfaction questionnaire was developed by Bolliger and Halupa in 2012 based on the Online Course Satisfaction Survey (OCSS) [29]. It consisted of 24 items categorised into the following subscales: instructor, technology, course setup, interaction, outcomes and overall satisfaction. The questionnaire used a five-point Likert scale, ranging from 1 (strongly disagree) to 5 (strongly agree). The faculty satisfaction questionnaire, the Online Instructor Satisfaction Questionnaire (OISQ), was developed by Bolliger and Wasilik in 2009 [5]. It consisted of 28 items, categorised into the following subscales: student, instructor and institution. The questionnaire used a four-point Likert scale, ranging from 1 (strongly disagree) to 4 (strongly agree). The two questionnaires showed evidence of validity, reliability and internal consistency of the instrument’s subscales in assessing satisfaction with online learning, using Cronbach’s alphas of 0.91 and 0.85 for students and faculty, respectively [21,30]. The questionnaires were piloted, and the Cronbach’s alphas were 0.89 and 0.84 for students and faculty, respectively. Some items were modified slightly based on the feedback from respondents to improve clarity.

Questionnaires were sent to expectant participants by email along with the study information sheet. Participants were informed that participation in the study was voluntary and that they could withdraw from the study at any time without any consequences. Online consent was received from all participants, and they were provided with contact information if they wanted to clarify doubts or ask questions. All data were coded to ensure anonymity.

SPSS was used for statistical analysis. Descriptive statistics were used to analyse the responses to the satisfaction scales. In the students’ satisfaction questionnaire, six items were recoded, where 5 = strongly disagree and 1 = strongly agree. For the faculty questionnaire, eleven items were recoded, where 4 = strongly disagree and 1 = strongly agree.

An independent sample *t*-test was used to compare the mean scores of the satisfaction subscale with gender and previous experience among faculty and students. The significance level was set at a *p*-value of less than .05. Student and faculty responses to the open-ended questions were analysed using the thematic analysis method.

Responses to open-ended questions for both students and faculty were analysed thematically, with MHT and WE undertake qualitative data analysis. The authors took the following steps, which were originally developed by Virginia Braun and Victoria Clarke [31] for conducting thematic analysis [32,33]: they familiarised themselves with the data, created the codes, generated the themes, and then reviewed the themes. The study was approved by the Research Ethics Committee at the institution (REC-20-04-26-01).

## Results

Three hundred and fifty-eight responses out of 370 were received from students, a response rate of 97.8%, comprised of 335 (93.6%) female and 23 (6.4%) male students. The majority of students (71.8%, n = 257) had no previous experience with online learning (Table 1). Students attended an average of four online courses during the pandemic, compared to one course before the pandemic.

**Table 1.** Demographic characteristics of participants

		Students		Faculty	
		N	%	N	%
College	Dental Medicine	63	17.6	11	15.7
	Pharmacy	94	26.3	8	11.4
	Health Sciences*	163	45.5	37	52.9
	Medicine	38	10.6	14	20.0
Gender	Male	23	6.4	37	52.9
	Female	335	93.6	33	47.1
Previous Experience	Yes	101	28.2	12	17.1
	No	257	71.8	58	82.9

* Health sciences include medical laboratory, nursing, physiotherapy, dietetics, and medical imaging

Of the students, 68.7% (n = 246) were less satisfied with online learning, and 41.6% (n = 149) would not recommend the online learning experience to others. Nevertheless, students were satisfied with the communication during online learning (60.9%, n = 218), and almost half the students (47.5%, n = 170) were satisfied with the flexibility afforded during online learning. Challenges faced by students were the time taken to download learning materials (35.2%, n = 126), and 34.4% (n = 123) were dissatisfied with collaborative activities during online learning (Table 2).

**Table 2.** Student satisfaction

					SD/D	N	SA/A
		M	SD	N	% (N)	% (N)	% (N)
Instructor	There was clear communication of class assignments	3.52	1.06	358	18.4% (66)	21.5% (77)	60.1% (215)
	Evaluation, test and feedback were given on time.	3.13	1.21	358	32.4% (116)	21.8% (78)	45.8% (164)
	I felt a part of the class and belonged to the online session.	3.58	1.03	358	12.0% (43)	29.1% (104)	58.9% (211)
	I am satisfied with faculty accessibility and availability. (Recoded)	2.81	1.12	358	38.8% (139)	33.5% (120)	27.7% (99)
Technology	I am satisfied with online discussion forums.	3.18	1.16	358	27.7% (99)	27.4% (98)	45.0% (161)
	I am satisfied with online communication including email and announcements.	3.56	1.16	358	17.9% (64)	21.2% (76)	60.9% (218)
	Blackboard LMS is user-friendly.	3.56	1.11	358	15.4% (55)	23.2% (83)	61.5% (220)
	I am satisfied with the download duration of learning resources. (Recoded)	3.05	1.21	358	31.3% (112)	33.5% (120)	35.2% (126)
Setup	I am satisfied with the number of online sessions.	3.27	1.17	358	20.7% (74)	32.1% (115)	47.2% (169)
	Online courses offered flexible timing.	3.19	1.23	358	29.1% (104)	23.5% (84)	47.5% (170)
	I am satisfied with the self-directed responsibilities assigned to me. (Recoded)	3.01	1.13	358	30.4% (109)	37.2% (133)	32.4% (116)
	I enjoyed working on projects during online learning.	3.19	1.26	358	28.2% (101)	29.6% (106)	42.2% (151)
Interaction	I am satisfied with the quality of interaction between me, the faculty and peers.	3.15	1.14	358	28.2% (101)	27.9% (100)	43.9% (157)
	I am satisfied with collaborative activities during online learning. (Recoded)	3.02	1.13	358	30.7% (110)	34.9% (125)	34.4% (123)
	I can relate my level of understanding to other students’.	3.33	0.99	358	16.2% (58)	42.2% (151)	41.6% (149)
	I am comfortable with participating in online sessions.	3.16	1.20	358	26.8% (96)	31.6% (113)	41.6% (149)
Outcome	I am satisfied with the level of required effort in online course.	3.09	1.33	358	30.7% (110)	24.3% (87)	45.0% (161)
	I am satisfied with my performance in online course. (Recoded)	3.17	1.24	358	29.9% (107)	29.3% (105)	40.8% (146)
	I will be satisfied with my final grade.	2.99	1.17	358	31.0% (111)	34.6% (124)	34.4% (123)
	I am able to apply what I learned in this online course.	3.08	1.19	358	29.6% (106)	28.8% (103)	41.6% (149)
Overall satisfaction	I will recommend this online learning experience to others.	2.81	1.29	358	41.6% (149)	22.6% (81)	35.8% (128)
	I am more satisfied with online learning compared to face-to-face sessions. (Recoded)	3.94	1.14	358	10.9% (39)	20.4% (73)	68.7% (246)
	My satisfaction level encourages me to register in other available online						
courses, such as online summer courses.	2.78	1.39	358	41.1% (147)	26.3% (94)	32.7% (117)	
	Overall, I am satisfied with this course.	3.04	1.24	358	30.7% (110)	27.9% (100)	41.3% (148)

SD = strongly disagree; D = disagree; N = neutral; A = agree; SA = strongly agree

The subscales of student satisfaction revealed a mean score of less than 3.4 out of 5 in all subscales. The instructor subscale yielded the highest mean score (M = 3.36 ± 0.82), followed by the technology subscale (M = 3.31 ± 0.88) (Table 3). The correlation between the overall satisfaction and subscales among students reveals a significant correlation between the overall satisfaction of students with technology (r = 0.615, p = .000) and interaction (r = .665, p = .000) (Table 4).

**Table 3.** Subscales of students’ and faculty satisfaction

	Subscale	M	SD
**Student satisfaction**	Instructor	3.36	.82
	Technology	3.31	.88
	Setup	3.16	.87
	Interaction	3.15	.82
	Outcomes	3.00	.91
	Overall	2.67	1.05
**Faculty satisfaction**	Student	2.66	.19
	Instructor	2.54	.28
	Institution	2.13	.35
	Overall	2.79	.45

**Table 4.** Correlation between the overall satisfaction and satisfaction subscales among students

	1	2	3	4	5	6	7
Instructor Satisfaction	-						
Technology Satisfaction	0.64						
Setup Satisfaction	0.578	.585**					
Interaction Satisfaction	0.619	.567**	0.712				
Outcome Satisfaction	0.559	.531**	0.675	.696**			
Overall Satisfaction	0.564	.615**	0.681	.665**	0.742		
Total Satisfaction Score	0.788	.791**	0.847	.851**	0.845	.863**	-

_**_ Correlation is significant at the 0.01 level (2-tailed).

_*_ Correlation is significant at the 0.05 level (2-tailed).

An independent *t*-test was conducted to compare student satisfaction with previous experience in online learning. The results suggest that previous experience in online learning does not affect satisfaction, as there were no statistically significant differences between students who had previous experience (75.5 ± 17.17) compared to students with no experience (77.07 ± 13.48), t(150.88) = .86, p = .394. Similarly, the results suggest that gender does not affect student satisfaction, as there was no statistically significant difference between male (76.17 ± 18.94) and female students (76.64 ± 14.30), t(356) = .15, p = .88

Seventy faculty responses were received, giving a response rate of 86.4%; 52.9% (n = 37) were men, and 82.9% (n = 58) had no previous experience teaching exclusively online (Table 1). Of the faculty, 62.9% (n = 44) were more satisfied with online teaching than the face-to-face method, and 92.9% (n = 65) reported that students were more enthusiastic about online learning than traditional learning (Table 5).

**Table 5.** Satisfaction among faculty

		M	SD	N	SD N (%)	D N (%)	A N (%)	SA N (%)
Students	Students interaction is higher in online compered to face-to-face courses.	3.09	.58	70	0 (0.0%)	9 (12.9%)	46 (65.7%)	15 (21.4%)
	Online learning is more flexible than face-to-face learning.	2.80	.71	70	2 (2.9%)	20 (28.6%)	38 (54.3%)	10 (14.3%)
	Students are actively involved in their learning during online courses.	2.50	.81	70	7 (10.0%)	28 (40.0%)	28 (40.0%)	7 (10.0%)
	Online teaching lead to missing face-to-face contact with students. (Recoded)	2.84	.82	70	4 (5.7%)	18 (25.7%)	33 (47.1%)	15 (21.4%)
	Students are communicating actively with me regarding online course queries.	3.33	.60	70	0 (0.0%)	5 (7.1%)	37 (52.9%)	28 (40.0%)
	I can login into my online course any time.	2.39	.62	70	3 (4.3%)	39 (55.7%)	26 (37.1%)	2 (2.9%)
	Students are more enthusiastic in online learning than traditional learning.	3.33	.60	70	0 (0.0%)	5 (7.1%)	37 (52.9%)	28 (40.0%)
	I am satisfied with online communication tools.	2.80	.71	70	1 (1.4%)	23 (32.9%)	35 (50.0%)	11 (15.7%)
	I am satisfied with my ability to provide feedback to my students in online course.	2.40	.80	70	9 (12.9%)	29 (41.4%)	27 (38.6%)	5 (7.1%)
	The interaction of students with faculty and course content is passive. (Recoded)	3.49	.63	70	0 (0.0%)	5 (7.1%)	26 (7.1%)	39 (55.7%)
	It is appreciated that students can access online course materials universally.	3.10	.70	70	0 (0.0%)	14 (20.0%)	35 (50.0%)	21 (30.0%)
	The participation of students in discussions in online learning is lower than in face to face. (Recoded)	2.90	.76	70	0 (0.0%)	24 (34.3%)	29 (41.4%)	17 (24.3%)
	Online teaching prevents me from knowing students compared to face-to-face. (Recoded)	2.43	.91	70	10 (14.3%)	30 (42.9%)	20 (28.6%)	10 (14.3%)
	Online teaching provides opportunity to students to continue their study during COVID-19 pandemic	2.91	.88	70	5 (7.1%)	15 (21.4%)	31 (44.3%)	19 (27.1%)
	It is a challenge to motivate students in online learning than in traditional teaching. (Recoded)	2.26	1.09	70	20 (28.6%)	25 (35.7%)	14 (20.0%)	9 (12.9%)
Instructor	I used less resources in online teaching compared to traditional teaching. (Recoded)	3.14	.57	70	0 (0.0%)	7 (10.0%)	46 (65.7%)	17 (24.3%0
	I use reliable technology for online teaching.	3.31	.77	70	1(1.4%)	10 (14.3%)	25 (35.7%)	34 (48.6%)
	Controlling students in the online environment is not a problem for me.	2.83	.65	70	0 (0.0%)	22 (31.4%)	38 (54.3%)	10 (14.3%)
	Online teaching requires me to be more innovative in using online resources. (Recoded)	2.77	.78	70	6 (8.6%)	13 (18.6%)	42 (60.0%)	9 (12.9%)
	Online teaching is frustrating due to technical difficulties. (Recoded)	3.24	.80	70	3 (4.3%)	7 (10.0%)	30 (42.9%)	30 (42.9%0
	Students use a range of resources in online learning than in face-to-face.	3.19	.72	70	1 (1.4%)	10 (14.3%)	34 (48.6%)	25 (35.7%0
	Technical difficulties do not discourage me from online teaching.	2.63	.88	70	7 (10.0%)	24 (34.3%)	27 (38.6%)	12 (17.1%)
Institution	I have a higher workload when teaching an online course as compared to the traditional one. (Recoded)	3.66	.54	70	0 (0%)	2 (2.9%)	20 (28.6%)	48 (68.6%)
	It takes me longer to prepare for an online course on a weekly basis than for a face-to face course. (Recoded)	3.23	.59	70	0 (0%)	6 (8.6%)	42 (60%)	22 (31.4%)
	I receive fair reward for online teaching.	3.30	.68	70	1 (1.4%)	6 (8.6%)	34 (48.6%)	29 (41.4%)
	I am worried about gaining lower course evaluations in an online course compared to face-to face course. (Recoded)	2.90	.68	70	2(2.9%)	14(20.0%)	43 (61.4%)	11 (15.7%)
Overall Satisfaction	Online teaching encourages me to teach my next courses using online approach.	2.84	.67	70	2 (2.9%)	16 (22.9%)	43 (61.4%)	9 (12.9%)
	I am more satisfied with online teaching compared to face-to face methods.	2.73	.79	70	4 (5.7%)	22 (31.4%)	33 (47.1%)	11 (15.7%)

SD = strongly disagree; D = disagree; A = agree; SA = strongly agree

The areas of most dissatisfaction reported by faculty were higher workload (97.1%, n = 68), longer preparation time (91.4%, n = 64), and technical problems (85.7%, n = 60) (Table 3). The main challenge reported by faculty was technical difficulties (85.8%, n = 60), and 65.7% (n = 46) thought that student participation in online discussions was lower than face-to-face. Moreover, 55.3% (n = 38) were not satisfied with their ability to provide feedback to students during online learning (Table 5).

Evaluating the overall faculty satisfaction with subscales revealed that all subscales had a mean score of less than 2.8 out of 4. The student subscale yielded the highest mean score (M = 2.66 ± 0.19) of all subscales (Table 3). Nevertheless, the correlation between the overall satisfaction score and satisfaction subscales among faculty did not show any significance.

An independent *t*-test was conducted to compare faculty satisfaction with previous experience with online learning. The results suggest that previous experience did not affect satisfaction, as there was no statistically significant difference between faculty with previous experience (83.5 ± 11.37) and faculty with no experience (82.09 ± 7.71), t(68) = .530, p = .073. Similarly, the results suggest that gender does not affect faculty satisfaction, as there was no statistically significant difference between males (82.00 ± 9.41) and females (82.70 ± 7.12), t(68) = .35, p = .25.

An independent-samples *t*-test was conducted to compare satisfaction scores between faculty and students. The results suggest statistically significant differences between students (76.61 ± 14.61) and faculty (82.33 ± 8.36), t(164) = 5.71, p = .000.

## Thematic analysis of the open-ended questions

One hundred twenty-six students and fifteen faculty responded to the open-ended questions. Most of the students reported that the main challenges during online learning were technical difficulties and support. The second reported difficulty was staying on-screen for a long time. Time zone differences were also reported by students residing outside the country. Some students stated that online learning was stressful, as most students were studying in a completely different environment that was not prepared for education. To increase satisfaction and future improvement, students suggested a combination of synchronous and asynchronous interactions. Most students also suggested incorporating other applications to engage them in learning, such as polls and gaming. Fewer students stressed the importance of office hours and academic advising. Students also thought proper communication before sessions would make them well-prepared. Finally, they stated that more online discussion accompanied by timely feedback from instructors would enhance their learning.

The most significant challenge faced by faculty was the preparation for online teaching. Most of the faculty reported colossal effort and time devoted to online teaching compared to face-to-face instruction. The faculty were very creative in teaching laboratory sessions, which was time-consuming and mentally exhausting. Similarly, the second reported challenge was technical difficulties; the faculty agreed on the importance of training and IT support. One faculty member also suggested incentives and rewards.

**Figure 1.**
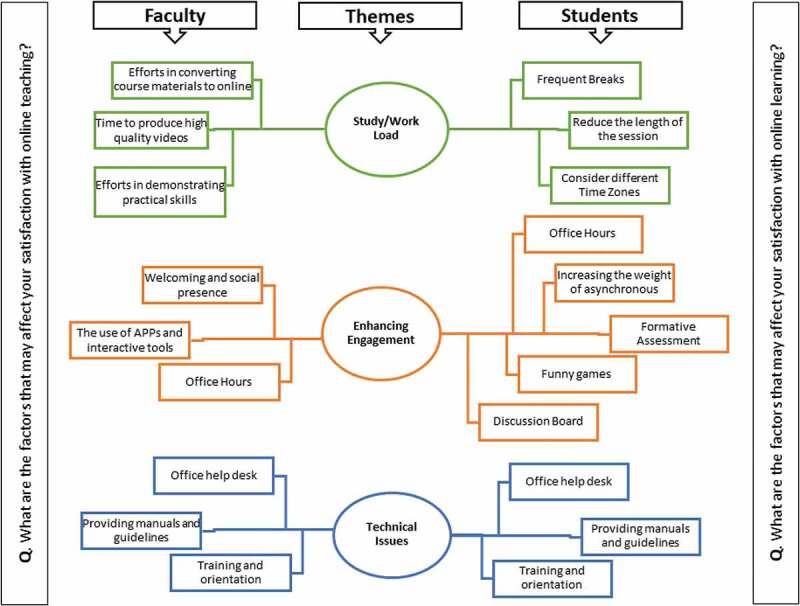
Thematic Network for analysis of open ended questions for students and faculty

The thematic network illustrates the three key themes and demonstrates the subthemes for both students and faculty (Figure 1). The network has three levels of themes: study-load and workload, enhancing engagement, and technical issues. Finally, the defined themes were named SWEET (Figure 1).

## Discussion

Student and faculty satisfaction with online learning is related to several factors, such as content, user interface, learning community, and learning performance [34]. Faculty satisfaction also substantially impacts online course outcomes [35]. To the best of our knowledge, no study has measured both faculty and student satisfaction simultaneously during the new normal; early studies measure either student or faculty satisfaction with online learning [26–28]. Therefore, validated, pretested tools [21,30] adapted to the current context were employed to report both faculty and student satisfaction during the new normal.

In the current study, students were satisfied with the communication and flexibility afforded during online learning. Interaction and technology were the greatest challenges reported by students along with engagement in collaborative activities during online learning. Faculty were satisfied with the communication and communication tools used during online learning. The areas of most dissatisfaction reported by faculty were higher workload, longer preparation time, and technical problems. There were statistically significant differences between student and faculty satisfaction.

The current study revealed that more than two-thirds of the students were less satisfied with online learning. This finding supports previous studies conducted in the USA, which reported that students were dissatisfied with online learning compared to face-to-face instruction [36,37]. Our findings might be due to personality, self-efficacy, and expectations related to the design and delivery of online learning and teaching, as reported in a previous study [38]. Another factor for decreased satisfaction could be attributed to the sudden shift to online delivery of the curriculum due to COVID-19, in which there was no adequate time for preparation, accompanied by the stressful working conditions of the pandemic itself [39].

The current study shows that students’ overall satisfaction correlated with technology satisfaction, and the most reported area of dissatisfaction was related to accessibility and availability of the instructor. In the current study, one-third of students were also dissatisfied with the amount of time taken to download resources. Many authors have reported that technical difficulties could lead to an ineffective learning experience, even using the best-designed online course. The same authors also reported that technology-related factors might impact student and faculty satisfaction with online education, including the level of technical support they can rely on and the user-friendliness of the technological infrastructure of their courses [40]. Recent studies have reported that the current generation of students has less tolerance for delays in the time taken to download resources. It has also been reported that they expect 24/7 availability of the faculty to respond to their late emails, calls and comments on online discussions [41].

The convenience and flexibility of online classes were found to be linked with student and faculty satisfaction [42,43]. In the current study, students agreed they were satisfied with the flexibility offered during online learning, and more than three-quarters of faculty thought it was valuable for students to have access to online courses worldwide. Nevertheless, due to the difference in zone time, students residing outside the country were not satisfied with time. These findings could be attributed to students’ family support in pursuing their learning remotely. It has been reported that health concerns and family obligations are essential inspirations for pursuing further online courses [44,45].

Several researchers have reported that most areas of faculty dissatisfaction are linked to workload, student engagement issues, and time spent preparing teaching materials [46,47]. The present study supports these early studies, where faculty reported a higher workload, difficulty motivating students in an online environment, and taking longer to prepare for an online course. Institutional support is essential for overcoming the aforementioned problems and has been recommended by many authors [46,47]. This support could be in the form of allowing sufficient time and compensative incentives for faculty [48]. Zheng et al. (2016) and Martin et al. (2018) explained that organisational policies regarding online learning could influence faculty satisfaction [24,49]. Some faculty members believe that online learning provides more flexibility, while others view online teaching as time-consuming and rigorous labour, which quickly leads to burnout [50–52].

Engaging students in online learning was one of the difficulties faced by faculty members. Students suggested formative assessment and online polls after sessions to give feedback to increase satisfaction and engagement. Feedback during online learning is essential to enhancing the achievement of learning outcomes [14], and effective feedback should be timely to enhance students’ learning and help them monitor their progress [53].

In the current study, students and faculty reported they were satisfied with the communication and communication tools they used during online learning. Tennyson and Hsia (2010) found that student–faculty interaction sustains a supportive learning environment, improves students’ performance, and elevates student satisfaction [54]. In contradiction, the lack of interaction with faculty, unclear learning expectations, and vague evaluation criteria lead to student dissatisfaction [55,56]. Moreover, Cidral et al. (2018) concluded that the instructor’s availability was crucial for the triumph of online learning [57]. Therefore, students might have adequate experience and better satisfaction in online classes when institutions provide sufficient online resources and technical support to enhance student-instructor interaction.

The current study demonstrates that students and faculty considered feedback during online learning to be useful and timely. Nevertheless, students suggested that adding online office hours would be beneficial to communication with their teachers. Effective feedback and communication from faculty compensate for the lack of face-to-face interaction and engage students in online learning [58]. Informal feedback has also been associated with enhancing communication among peers and faculty as they offer ways to maintain or improve performance [59].

More than one-third of the students (41.6%) were satisfied with peer interaction and 45% of students were satisfied with online discussions, compared to 65.7% of faculty who thought that the participation of students in online discussions is lower than in face-to-face classes. Students also suggested that small group discussions and fun gaming activities increase student interaction and engagement. These findings agree with previous studies that peer and collaborative learning led to better achievement and student engagement in online learning [24]. Peer interaction provides opportunities to improve learning and enhances student experiences [60]. Online discussion also plays a significant role in online learning success [61]. Discussion is an active learning process that replaces passive listening and encourages students to engage in content with conversation and reflections. Using interactive multimedia facilitates active learning [62]. It also encourages authentic learning, problem-solving skills, and reflection to develop new knowledge [63].

The findings of the current study could be beneficial for planning, designing and delivering online learning activities, and it could increase student and faculty satisfaction with online courses and, consequently, the quality of learning. Correspondingly, it would be advantageous in increasing student engagement. To increase satisfaction and future improvement, the study recommends a combination of synchronous and asynchronous online approaches, incorporating different applications with the learning management systems used to engage students in online learning. Constructive and timely feedback on student performance is essential to enhancing their satisfaction with online learning. Training of faculty and orientation of students in addition to IT support will improve the satisfaction of both students and faculty. Finally, institutional support, including organisational policy, incentives and faculty development, will enhance faculty satisfaction with online teaching. The findings of the study may be of interest to college leaders and educational development, as satisfaction is a key differentiator in a competitive marketplace and crucial for improvement.

One of the current study’s strengths is that it reports satisfaction with online learning from two perspectives: student and faculty. This study highlighted common factors affecting student and faculty satisfaction with online learning in the new normal. The study further compared the satisfaction of both students and faculty. The limitation of this study is the use of a self-assessment questionnaire. Further analysis is required to provide an in-depth exploration of factors affecting satisfaction. The use of a comprehensive qualitative approach, including a focus group discussion and interviews, might be of help.

## Conclusion

Online learning has been a useful and practical tool for curriculum delivery during COVID-19. Effective communication and flexibility afforded during online learning have been linked with increasing student satisfaction. Challenges facing students were the long duration of learning sessions and technology. On the other hand, technical assistance and students’ enthusiasm enhanced faculty satisfaction with online teaching. Higher workload, longer preparation time, and technical problems were challenges reported by faculty.
